# Optimizing Timing for Respiratory Syncytial Virus Prevention Interventions for Infants

**DOI:** 10.1001/jamanetworkopen.2025.22779

**Published:** 2025-07-23

**Authors:** Danielle Nguyen, Haeseon Lee, Andrew T. Pavia, Richard E. Nelson, Matthew Samore, Nathorn Chaiyakunapruk

**Affiliations:** 1 Department of Pharmacotherapy, College of Pharmacy, University of Utah, Salt Lake City; 2 Division of Pediatric Infectious Diseases, University of Utah, Salt Lake City; 3 IDEAS (Informatics, Decision-Enhancement, and Analytic Sciences) Center, Veterans Affairs Salt Lake City Health Care System, Salt Lake City, Utah; 4 Division of Epidemiology, School of Medicine, University of Utah, Salt Lake City

## Abstract

**Importance:**

The Advisory Committee on Immunization Practices and the Centers for Disease Control and Prevention recommend seasonal administration of maternal vaccine (MV) or nirsevimab to protect infants in the first year of life from respiratory syncytial virus (RSV) infections. Differences in uptake, costs, and efficacy between these agents may affect cost-effectiveness depending on the timing of administration.

**Objective:**

To evaluate the clinical outcomes and cost-effectiveness of no intervention, MV, and nirsevimab administration during the entire RSV season and separately for each monthly birth cohort from October through February.

**Design, Setting, and Participants:**

This economic evaluation used a Markov model to analyze cost-effectiveness from a societal perspective, applying a willingness-to-pay threshold of $150 000 per quality-adjusted life-year (QALY). Participants included infants born in the US during the RSV season. Data were accrued from October 2023 to June 2024 and analyzed from July 2024 to May 2025.

**Interventions:**

MV, nirsevimab administration, or no intervention.

**Main Outcomes and Measures:**

The primary outcome was the incremental cost-effectiveness ratio (cost per QALY gained). Clinical outcomes included the number of hospitalizations, outpatient infections, and deaths averted. Probabilistic sensitivity analyses were conducted.

**Results:**

An estimated monthly birth cohort of 299 277 infants was included in the analysis. Compared with no intervention, MV was cost-saving for infants in the October, November, and December cohorts and cost-effective in the January but not the February cohorts ($504 517/QALY). For infants born in the combined cohort (October-February), MV was cost-effective at $19 562/QALY. Compared with MV, nirsevimab was cost-effective only in October ($67 178/QALY) and November ($88 531/QALY). During the RSV season, MV was projected to avert 45 558 outpatient visits, 7154 hospitalizations, and 12 deaths; nirsevimab was projected to avert 92 265 outpatient visits, 11 893 hospitalizations, and 19 deaths. The probability of no intervention being cost-effective during the RSV season was 19.8%; MV, 62.2%; and nirsevimab, 18.0%.

**Conclusions and Relevance:**

In this economic evaluation of RSV prevention interventions, administration of MV in the first 4 months of and throughout the viral season could be cost-effective. Across the entire RSV season, nirsevimab was cost-effective compared with MV only in October and November. Intervention use may be optimized by restricting administration to select months. Further study is needed to assess transmission dynamics to refine cost-effectiveness outcomes.

## Key Points

**Question:**

What is the cost-effectiveness of no intervention, maternal vaccination, and nirsevimab administration during the overall respiratory syncytial virus (RSV) season and for each monthly birth cohort?

**Findings:**

In this economic evaluation of an estimated monthly birth cohort of 299 277 infants, maternal vaccination was cost-effective compared with no intervention ($19 562 per quality-adjusted life-year), but these results were driven by infants born earlier in the season. Compared with maternal vaccination, nirsevimab was cost-effective only in October and November, at $67 178 and $88 531 per quality-adjusted life-year, respectively.

**Meaning:**

The seasonal nature of RSV and the waning immunogenicity of these interventions suggest that earlier administration could optimize clinical and economic outcomes.

## Teaser

This economic evaluation assesses the cost-effectiveness and clinical outcomes of maternal vaccination and nirsevimab administration compared with no intervention at different times during the respiratory syncytial virus (RSV) season among US infants.

## Introduction

In the US, respiratory syncytial virus (RSV) is the most common cause of pediatric respiratory infections, resulting in 2.1 million outpatient visits and 80 000 hospitalizations annually in children younger than 5 years.^1,2^ Infants younger than 1 year have the highest infection risk, and RSV is the leading cause of hospitalization.^1,3^ Prior to 2023, the only US Food and Drug Administration–approved intervention for preventing severe RSV infection was palivizumab, a monoclonal antibody requiring monthly injections, which is relatively costly and limited to high-risk infants (10% of infants).^4,5,6^ This leaves the remaining 90% of infants susceptible, and at least 75% of RSV-related hospitalizations occur in those without risk factors for severe disease.^6,7,8,9,10^

A maternal bivalent prefusion F vaccine (MV)^11^ and nirsevimab, a long-acting monoclonal antibody,^12^ were approved in 2023 to prevent RSV infection in the general infant population. Both are recommended by the Advisory Committee on Immunization Practices and the Centers for Disease Control and Prevention for seasonal administration. MV can be administered to pregnant women at a gestational age (GA) of 32 to 36 weeks from September through January, while nirsevimab can be administered to the infant after birth if MV was not used. Both agents are viable options for RSV protection for infants born from October through February but differ in uptake, costs, efficacy, and waning protection, which may influence cost-effectiveness based on timing of administration. To our knowledge, no US-based economic evaluation has assessed both agents in the same analysis while considering timing. This study evaluates the cost-effectiveness and clinical outcomes of MV and nirsevimab compared with no intervention when (1) administered during different months throughout the RSV season or (2) when considering immunization from October through February.

## Methods

In this economic evaluation, we followed recommendations from the Second Panel on Cost-Effectiveness in Health and Medicine and reported results in accordance with Consolidated Health Economic Evaluation Reporting Standards (CHEERS) guidelines (eTable 1 in Supplement 1).^13^ Because this was not human participants research, institutional review board approval and informed consent were not required per the Common Rule.

### Model Overview

**Figure 1.**
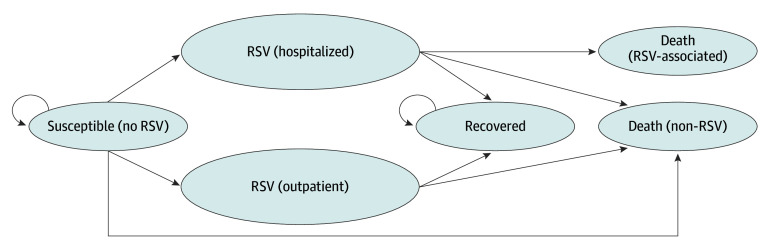
Markov Model RSV indicates respiratory syncytial virus infections.

We modeled the course of an infant’s first RSV season using a Markov model with 1-month cycles. All infants started in the no RSV (susceptible) state and could remain susceptible to RSV, contract RSV, or die from other causes (Figure 1). Immunized infants had lower RSV risks than nonimmunized infants. If RSV was contracted, the infection could be managed in the outpatient or inpatient setting. After infection, infants would be recovered, which assumed immunity. Reinfections were assumed to not significantly contribute to costs or disutility, as they are mild and uncommon.^14^ Infection risk changed monthly based on infant age, RSV seasonality, and immunization efficacy.

The cohort population included newborns eligible for either MV or nirsevimab.^5,15^ A monthly birth rate of 299 277 was assumed (Table 1).^16^ Only infants born at or after a GA of 32 weeks were included, as those born prior would be ineligible for MV.^48,49^ Infants born earlier than 2 weeks post MV were assumed to receive additional nirsevimab. Six different birth cohorts were evaluated: those born during the RSV season and each month between October and February. The time horizon was 6 months because minimal reduction in infections is expected after the first RSV season. The model was developed using Excel, version 2408 (Microsoft 365; Microsoft Corporation). Additional details on the input parameters are found in eTable 2 in Supplement 1.

**Table 1.** Estimated Input Parameters

Parameter	Value (range)	Distribution	Source
**General**			
Birth rate per month, mean	299 277 (NA)	NA	Osterman et al,^16^ 2024
Lifetime expectancy, mean, y	77.5 (NA)	NA	Kochanek et al,^17^ 2022
RSV hospitalization incidence rate	Supplement 1 ^a^	β	McMorrow et al,^18^ 2024; Curns et al,^19^ 2024
RSV outpatient infection incidence rate	Supplement 1 ^a^	β	Gantenberg et al,^20^ 2022; McMorrow et al,^18^ 2024; Curns et al,^19^ 2024
**Clinical **			
Vaccination uptake, mean, %			
MV	58.4 (46.7-70.1)	β	Jarshaw et al,^21^ 2024
MV plus nirsevimab	8.5 (6.8-10.1)	β	Jacobson et al,^22^ 2025
Nirsevimab	79.8 (63.8-95.7)	β	Hill et al,^23^ 2023
Immunization hospitalization efficacy, mean, %a^b^			
MV	67.9 (34.5-84.3)	β	Kampmann et al,^24^ 2023
Nirsevimab	76.8 (49.4-89.4)	β	Muller et al,^25^ 2023
Immunization outpatient efficacy, mean, %^b^			
MV	48.2 (23.3-65.0)	β	Kampmann et al,^24^ 2023
Nirsevimab	78.0 (56.9-88.0)	β	Hammit et al,^26^ 2022; Muller et al,^25^ 2023
Duration of illness, mean, d			
Hospitalization	2.42 (1.94-2.90)	γ	Doucette et al,^27^ 2016
Infection	7 (5-10)	γ	Régnier,^28^ 2013; Eiland,^29^ 2009; Hodgson et al,^30^ 2020; Hak et al,^31^ 2024
Death, mean, %			
Inpatient RSV CFR	0.16 (0.04-0.90)	β	Curns et al,^19^ 2024; Doucette et al,^27^ 2016; Rha et al,^32^ 2020
Non-RSV death rate	0.045 (0.036-0.054)	β	Ely and Driscoll,^33^ 2023
**Costs**			
Caregiver productivity loss, mean, $			
Salary (per work week)	1302 (709-1531)	γ	US Bureau of Labor Statistics,^34^ 2024
Lifetime market productivity loss	1 219 675 (975 740-1 463 610)	γ	Grosse et al,^35^ 2019
Lifetime nonmarket productivity loss	696 817 (557 454-836 181)	γ	Grosse et al,^35^ 2019
Medical costs, $^c^			
RSV hospitalization, mean	20 566 (18 389-23 594)	γ	Curns et al,^19^ 2024; Rha et al,^32^ 2020; Bowser et al,^36^ 2022; Averin et al,^37^ 2024
RSV hospitalization, median	10 313 (9911-10 760)	γ	Curns et al,^19^ 2024; Rha et al,^32^ 2020; Bowser et al,^36^ 2022; Averin et al,^37^ 2024
Hospitalization transportation cost, mean	2.91 (2.32-3.49)	γ	Federal Highway Administration,^38^ 2021; US Department of Energy^39^ 2024; Weiss and Roemer,^40^ 2021
RSV outpatient, mean	588 (568-608)	γ	Curns et al,^19^ 2024; Rha et al,^32^ 2020; Bowser et al,^36^ 2022; Averin et al,^37^ 2024
RSV outpatient, median	387 (378-397)	γ	Curns et al,^19^ 2024; Rha et al,^32^ 2020; Bowser et al,^36^ 2022; Averin et al,^37^ 2024
Outpatient transportation cost, mean	2.52 (2.01-3.02)	γ	Federal Highway Administration,^38^ 2021; US Department of Energy^39^ 2024; Akinlotan et al,^41^ 2023
Drug costs, median, $			
Maternal vaccine	272 (230-307)	γ	Centers for Disease Control and Prevention,^42^ 2025
Nirsevimab	496 (415-516)	γ	Centers for Disease Control and Prevention,^42^ 2025
Administration cost	15.67 (12.99-18.35)	γ	Centers for Medicare & Medicaid Services,^43^ 2024
Utilities, mean			
RSV hospitalization	0.58 (0.41-0.75)	β	Glaser et al,^44^ 2022; Leidy et al,^45^ 2005; Régnier,^28^ 2013
RSV outpatient	0.78 (0.55-1.00)	β	Glaser et al,^44^ 2022; Leidy et al,^45^ 2005; Régnier,^28^ 2013
RSV post-hospitalization	0.78 (0.55-1.00)	β	Glaser et al,^44^ 2022; Leidy et al,^45^ 2005; Régnier,^28^ 2013
Lifetime	Supplement 1 ^d^	β	Jiang et al,^46^ 2021; Protecting Health Care for All Patients Act of 2023^47^

Abbreviations: CFR, case fatality ratio; NA, not applicable; RSV, respiratory syncytial virus.

^a^ eFigure 1 and eTable 3 in Supplement 1 show age-adjusted incidence and RSV seasonality.

^b^ This is reported for the initial efficacy. All values are reported in eTable 4 in Supplement 1.

^c^ Costs are inflated to 2024.

^d^ See eTable 5 in Supplement 1.

### Inputs

#### Incidence of RSV

Annual incidences for RSV hospitalization and medically attended RSV outpatient infection Monthly incidences for RSV hospitalization and medically attended RSV outpatient are provided in eTable 3 in Supplement 1.^18,20^ Hospitalizations reflected inpatient-managed lower respiratory tract infections (LRTIs), while outpatient infections included LRTIs managed in the emergency department or outpatient settings without hospitalization.^20^ Incidence was age adjusted and modeled seasonally, using published New Vaccine Surveillance Network (NVSN) data from 2016 to 2020 for hospitalizations and 2005 to 2009 for outpatient infections (eFigure 1 and eTable 3 in Supplement 1).^18,19,20,50,51^ Post-2020 data were excluded due to atypical RSV trends during COVID-19 and the advent of nirsevimab and MV.

#### Death

The 30-day non-RSV death rate was estimated as 0.045% (range, 0.036%-0.054%) based on the National Vital Statistics System (Table 1).^33^ The inpatient RSV case fatality ratio (CFR) was assumed to be 0.16% (range, 0.04%-0.90%) (Table 1).^19,27,32^ RSV-related deaths were assumed to occur only during hospitalization. The non-RSV death rate was applied to the remaining health states, including outpatient-managed infections.

#### Immunization Efficacy

Efficacies were based on the MATISSE (Maternal Immunization Study for Safety and Efficacy) and MELODY trials.^24,25,26^ For RSV hospitalization, initial efficacy was 67.9% (95% CI, 34.5%-84.3%) for MV and 76.8% (95% CI, 49.4%-89.4%) for nirsevimab ^24,25,26^ (Table 1 and eTable 4 in Supplement 1). For RSV outpatient infections, the initial efficacy was 48.2% (95% CI, 23.3%-65.0%) for MV and 78.0% (95% CI, 56.9%-88.0%) for nirsevimab.^24,25,26^ For infants receiving MV and additional nirsevimab, immunization efficacy was based on nirsevimab, given the limited time for maternal antibody development. Efficacies against hospitalization were directly obtained from clinical trials, while those against outpatient management were calculated using medically attended LRTI and hospitalization data (eTable 4 and eMethods in Supplement 1). MV uptake was estimated to be 58.4% (range, 46.7%-70.1%) and nirsevimab uptake to be 79.8% (range, 63.8%-95.7%) based on prenatal influenza and birth-dose hepatitis B vaccine uptake (Table 1).^21,23^ Based on cohort data reporting dual intervention uptake, an estimated 8.5% of infants would require both due to birth within 2 weeks post MV.^22^

### Costs

MV and nirsevimab mean costs were $272 (range, $230-$307) and $496 (range, $415-$516), respectively, weighted by the US payer mix (Table 1).^42^ Administration mean costs were $15.67 (range, $12.99-$18.35).^43^ RSV-associated hospitalization and outpatient-managed mean infection costs for infants born at a GA of at least 32 weeks were estimated to be $20 566 (range, $18 389-$23 594) and $588 (range, $568-$608), respectively, based on weighted averages of inpatient care and outpatient care (including emergency department care). The mean hospitalization and outpatient care costs were used for the base-case analysis to reflect higher costs in infants with comorbidities (ie, congenital lung disease).^36,37^ A sensitivity analysis used median costs: $10 313 for hospitalization and $387 for outpatient-managed infections.^37^ All health care utilization costs were adjusted to reflect the GA distribution and payer mix of the US.^19,32,36^ Transportation costs were based on the price of gas to the hospital or the provider.^38,39,40,41^ Caregiver productivity loss was based on a mean weekly salary of $1302 (range, $709-$1531).^34^ Lifetime productivity losses due to RSV-associated death included market ($1 219 675 [range, $975 740-$1 463 610]) and nonmarket ($696 817 [range, $557 454-$836 181]) productivity.^35^ All costs were specific to the US and inflated to 2024 dollars.^52^

### Utilities

Utility for RSV hospitalization was estimated to be 0.58 (range, 0.41-0.75); for posthospitalization recovery, 0.78 (range, 0.55-1.00); and for outpatient-managed RSV infection, 0.78 (range, 0.55-1.00).^28,44,45^ Hospitalization was assumed to last a mean of 2.42 (range, 1.94-2.90) days (Table 1).^27^ Posthospitalization or outpatient-managed RSV infection recovery was assumed to be 7 (range, 5-10 days) days.^28,29,30,31^ Lifetime utility loss from RSV-related death was included (eTable 5 in Supplement 1).^17,46^

### Statistical Analysis

Data were analyzed from July 2024 to May 2025. The model was developed using Excel, version 2408 (Microsoft 365; Microsoft Corporation).

#### Base-Case Analysis

Clinical outcomes, quality-adjusted life-years (QALYs), and costs were assessed from a societal perspective for 6 birth cohorts: October, November, December, January, February, and all 5 months combined. For each cohort, hospitalizations, outpatient infections, and RSV-associated deaths for a national population were reported as the total number of cases, events averted, and the number needed to vaccinate to prevent an event. The primary outcome was the incremental cost-effectiveness ratio (ICER), the additional cost per QALY gained. Cost-effectiveness was assessed using a willingness-to-pay (WTP) threshold of $150 000/QALY, that is, the maximum amount society is willing to invest per additional QALY gained.^53,54,55^ Interventions with an ICER below this threshold were considered cost-effective. Both costs and QALYs were discounted at an annual rate of 3%, and costs are reported in 2024 US dollars.^13^

#### Sensitivity Analyses

Deterministic 1-way and probabilistic sensitivity analyses (PSAs) were performed to evaluate the robustness of our results. In the 1-way sensitivity analyses, each parameter was adjusted individually to its lowest and highest plausible values to assess its association with the ICER (Table 1). This approach detects parameters with the greatest influence. The results were presented as tornado diagrams, ranking parameters by descending association with the ICER. For the PSAs, we conducted 1000 Monte Carlo simulations in which all parameters were simultaneously varied within their respective distributions and ranges to capture the combined uncertainty (Table 1). Using a net monetary benefit approach, we calculated the probability of each intervention being cost-effective at various WTP thresholds. The PSA results are presented as a cost-effectiveness acceptability curve, which shows the probability of being cost-effective across a range of WTP thresholds. A threshold analysis was conducted to identify the price at which immunization would become cost-effective for the full RSV season, based on a WTP threshold of $150 000/QALY.

#### Scenario Analyses

Scenario analyses assessed the interventions under varying conditions: from a health care perspective, using the equal-value life-years gained (evLYG) approach, using median hospitalization and outpatient costs, and applying the friction cost method to estimate productivity, which would exclude productivity losses due to premature death. An evLYG-based analysis was conducted as a comparison with the standard QALY approach.^56^ Unlike QALYs, which adjust utility by age, the evLYG approach applies a fixed utility of 0.851, which is the mean utility for the US population, for each additional survival year.^47^ The evLYG equates the value of life extension equally regardless of current age. It was evaluated against a WTP threshold of $150 000/evLYG.^57^

## Results

### Base-Case Analysis

The administration of MV and nirsevimab for the birth cohort of 299 277 infants born in October reduced RSV-associated hospitalizations to 3111 and 1877 compared with no intervention, respectively.^16^ This corresponds to 1514 and 2748 events averted with numbers needed to vaccinate of 118 and 87, respectively (Table 2). Similar patterns in the reduction of outpatient visits and RSV-related deaths were observed. Improved clinical outcomes were seen after administration of MV and nirsevimab for all birth cohorts during the RSV season. With immunization across the combined October-February season, MV could avert 45 588 outpatient visits, 7154 hospitalizations, and 12 RSV-related deaths; nirsevimab could avert 92 265 outpatients visits, 11 893 hospitalizations, and 19 RSV-related deaths (Table 2).

**Table 2.** Estimated Clinical Effects of Maternal and Infant Immunization

RSV outcomes by birth month	Total No. of events			No. of events averted^a^			No. needed to vaccinate^b^		
	No intervention	MV	Nirsevimab	MV	Nirsevimab	Difference	MV	Nirsevimab	Difference
**Infections**									
Hospitalized									
October	4625	3111	1877	1514	2748	−1234	118	87	31
November	5122	3275	2045	1847	3077	−1230	96	78	19
December	4654	2913	1839	1741	2815	−1075	102	85	17
January	3320	2051	1302	1269	2018	−749	139	118	21
February	2028	1244	794	784	1235	−451	225	193	32
October to February	19 749	12 595	7856	7154	11 893	−4739	124	100	24
Outpatient									
October	46 625	32 961	19 190	13 665	27 436	−13 771	13	9	5
November	41 568	29 314	16 704	12 254	24 865	−12 610	15	10	5
December	32 131	22 567	12 671	9563	19 459	−9896	19	12	7
January	21 049	14 718	8173	6331	12 876	−6545	29	19	10
February	12 469	8694	4840	3775	7629	−3854	48	31	17
October to February	153 843	108 255	61 578	45 588	92 265	−46 677	20	13	7
**Deaths**									
October	7	5	3	2	4	−2	73 024	54 105	18 919
November	8	5	3	3	5	−2	58 494	47 465	11 029
December	8	5	3	3	5	−2	61 557	51 485	10 072
January	5	3	2	2	3	−1	84 089	71 534	12 555
February	3	2	1	1	2	−1	136 178	117 192	18 986
October to February	32	20	13	12	19	−8	75 448	61 360	14 088

Abbreviation: MV, maternal vaccination.

^a^ These are the events averted compared with no intervention.

^b^ Difference is measured as MV minus nirsevimab.

From a societal perspective, the administration of MV yielded 147 064 QALYs, with costs of $197 022 041 for infants born in October. Nirsevimab yielded 147 185 QALYs, with costs of $205 117 251 in the same period, and no intervention yielded 146 929 QALYs with costs of $205 453 982 . The total costs and QALYs for other months are provided in Table 3. At a WTP threshold of $150 000/QALY, compared with no intervention, MV was cost-saving for infants born in October, November, and December. MV was cost-effective for infants born in January ($124 032/QALY) but not in February ($504 517/QALY). Across the RSV season, MV was cost-effective with an ICER of $19 562/QALY. Compared with no intervention, nirsevimab was cost saving in October and November and cost-effective in December ($55 999/QALY) but not for January ($284 617/QALY) or February ($779 935/QALY). For the RSV season (ie, October through February), nirsevimab was cost-effective compared with no intervention ($128 753/QALY) (eTable 6 in Supplement 1). However, nirsevimab was cost-effective in October ($67 178/QALY) and November ($88 531/QALY) when compared with MV, with the ICER values increasing to $1 175 215/QALY in February, and an overall ICER of $268 590/QALY for the entire RSV season (Table 3). Incremental costs, QALYs, and ICERs for both the MV vs no intervention and nirsevimab vs MV comparisons are in Table 3.

**Table 3.** Model Estimates of a QALY-Based Cost-Effectiveness Analysis Associated With Infant and Maternal Immunization From a Societal Perspective

Societal Perspective	Intervention	Total costs, $	Total QALYs	Incremental cost, $^a^	Incremental QALYs^a^	ICER, Cost/QALY^a^
October	No intervention	205 453 982	146 929	Reference	Reference	Reference
	MV	197 022 041	147 064	−8 431 941	135	Dominant
	Nirsevimab	205 117 251	147 185	8 095 210	121	$67 178
November	No intervention	208 915 202	146 919	Reference	Reference	Reference
	MV	194 596 896	147 067	−14 318 306	148	Dominant
	Nirsevimab	204 914 619	147 183	10 317 723	117	$88 531
December	No intervention	179 329 533	146 980	Reference	Reference	Reference
	MV	172 776 682	147 111	−6 552 851	131	Dominant
	Nirsevimab	192 158 561	147 209	−19 381 879	98	$198 549
January	No intervention	124 456 363	147 095	Reference	Reference	Reference
	MV	136 032 856	147 188	11 576 493	93	$124 032
	Nirsevimab	170 027 957	147 255	33 995 101	67	$509 055
February	No intervention	75 274 842	147 199	Reference	Reference	Reference
	MV	104 054 075	147 256	28 779 234	57	$504 517
	Nirsevimab	150 763 757	147 296	46 709 681	40	$1 175 215
October to February	No intervention	793 429 922	735 121	Reference	Reference	Reference
	MV	804 482 550	735 686	11 052 628	565	$19 562
	Nirsevimab	922 982 145	736 128	118 499 595	441	$268 590

Abbreviations: ICER, incremental cost-effectiveness ratio; MV, maternal vaccination; QALY, quality-adjusted life-year.

^a^ MV is compared with no intervention; nirsevimab is compared with MV, the next best alternative.

### Scenario Analyses

The evLYG-based analysis produced results consistent with the base-case analysis (eTable 7 in Supplement 1). At a WTP threshold of $150 000/evLYG, MV was cost saving for infants born in October, November, and December. MV was cost-effective in January ($127 517/evLYG) but not in February ($518 831/evLYG). During the RSV season, MV was cost-effective ($20 068/evLYG). When compared with MV, nirsevimab was cost-effective in October ($68 534/evLYG) and November ($90 411/evLYG) but not the remaining months. The overall ICER was $274 402/evLYG for the entire RSV season (eTable 7 in Supplement 1).

When median hospitalization and outpatient costs were analyzed, MV was cost-effective for infants born in October ($72 664/QALY), November ($47 868/QALY), and December ($100 503/QALY) only and not for the RSV season (eTable 8 in Supplement 1). Nirsevimab was not cost-effective compared with MV in any month.

When the friction cost method was used to calculate productivity loss, MV was no longer cost-effective in January ($167 204/QALY) compared with no intervention, but results for nirsevimab remained similar to the base-case analysis (eTable 9 in Supplement 1). When a health care perspective was used and the value of productivity loss was no longer incorporated, MV remained cost-effective compared with no intervention for infants born in October ($123 469/QALY), November ($72 318/QALY), and December ($110 008/QALY) only (eTable 10 in Supplement 1). However, nirsevimab was not cost-effective for any month, even when compared with no intervention (eTable 6 in Supplement 1).

### Sensitivity Analyses

In the 1-way sensitivity analyses, the cost-effectiveness findings comparing MV with no intervention for the October, November, and December birth cohorts were robust at a WTP threshold of $150 000/QALY (eFigures 2 and 3 in Supplement 1). For the January and combined October through February birth cohorts, the results were influenced by vaccine efficacy, vaccine cost, infection incidence and duration, hospitalization cost, outpatient cost, discount rate, and inpatient RSV CFR. The cost-effectiveness findings comparing nirsevimab with MV were sensitive to the following factors at a WTP threshold of $150 000/QALY: for October, vaccine efficacy and drug cost; for November, vaccine efficacy, drug cost, inpatient RSV CFR, and nirsevimab uptake; for December, vaccine efficacy, inpatient RSV CFR, and drug cost; for January and February, inpatient RSV CFR; and for the combined October through February cohort, hospitalization vaccine efficacy and inpatient RSV CFR (eFigures 4 and 5 in Supplement 1).

**Figure 2.**
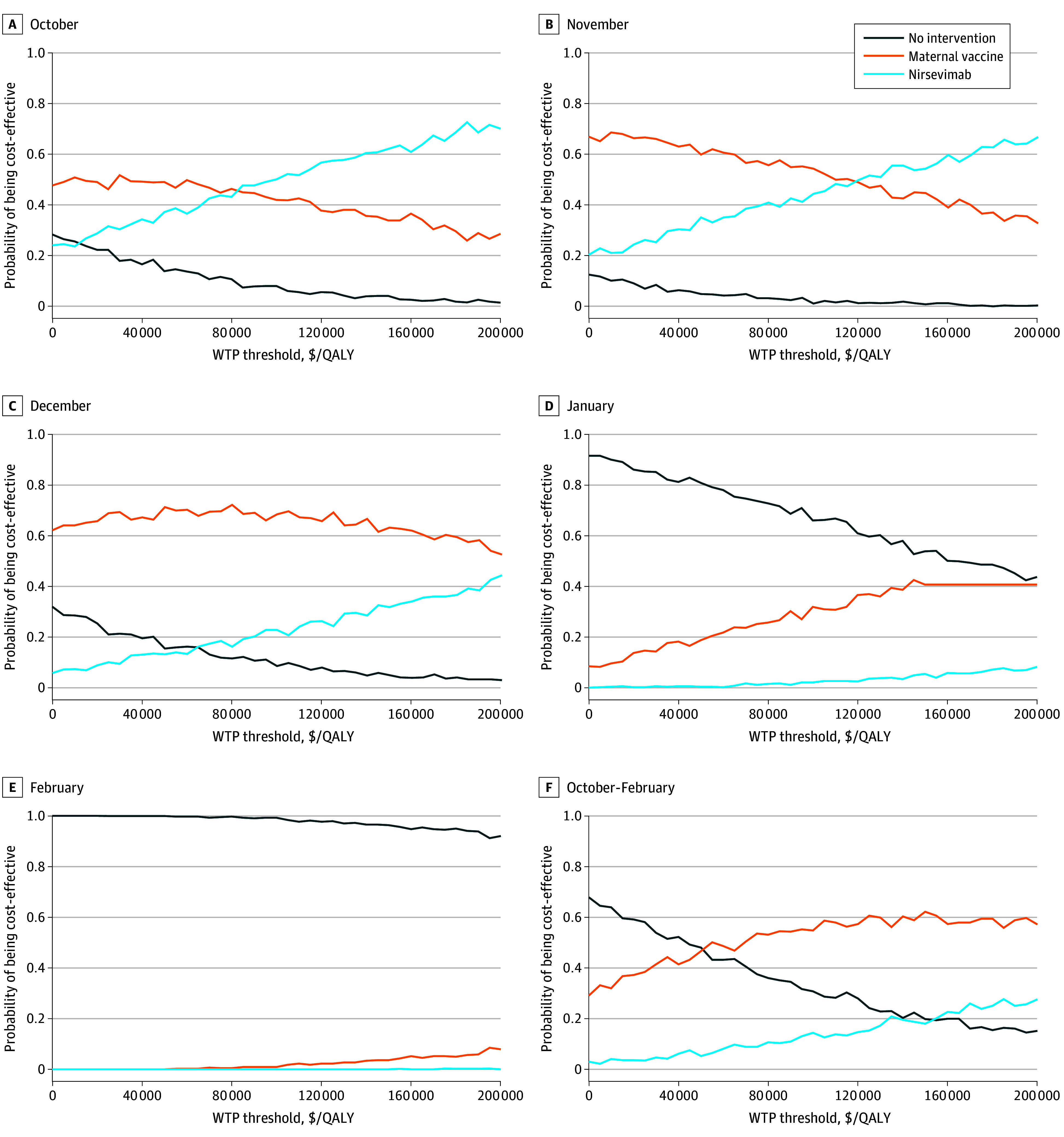
Cost-Effectiveness Acceptability Curves for Respiratory Syncytial Virus Prevention Interventions Data are stratified by month of birth. WTP indicates willingness to pay.

In the PSAs, the likelihood of a full vaccination program being cost-effective at a WTP threshold of $150 000/QALY was 62.2% for MV, 18.0% for nirsevimab, and 19.8% for no intervention (Figure 2). MV showed varying probabilities of cost-effectiveness by month: 33.8% for October, 44.8% for November, 63.2% for December, 40.7% for January, and 3.7% for February. For nirsevimab, these probabilities were 62.1% for October, 54.3% for November, 31.8% for December, 5.4% for January, and 0 for February. In the threshold analysis, the price of nirsevimab would need to decrease to $444 (10% reduction) to be cost-effective at $150 000/QALY from a societal perspective compared with MV when administered from October through February.

## Discussion

This economic analysis is one of the first, to our knowledge, to directly compare MV and nirsevimab within a cost-effectiveness framework, incorporating a month-by-month evaluation. In most US regions, RSV seasonality follows a predictable pattern, with onset in the fall, a winter peak, and offset in the spring.^58^ This seasonality was associated with the cost-effectiveness of RSV prevention strategies. Early administration may result in waning vaccine efficacy during the seasonal RSV peak, but late administration may prevent too few cases to be cost-effective. RSV risk also varies with age, further complicating the intervention timing.^18,19^ MV was cost-effective when administered throughout the RSV season, although this was driven by October, November, December, and January births. The November cohort demonstrated the lowest ICER due to the alignment of vaccine efficacy with the seasonal RSV peak. Nirsevimab was cost-effective compared with no intervention during the overall season and was cost-effective compared with MV in October and November. By accounting for age-specific RSV risk, birth timing, seasonal infection patterns, immunization efficacy, and associated costs, our model provides a comprehensive framework for evaluating these strategies on a month-by-month basis.

US-based economic evaluations comparing nirsevimab and MV remain limited.^59,60,61^ Hutton et al^59,60^ conducted separate cost-effectiveness analyses of nirsevimab and MV vs no intervention, reporting ICERs of $153 517/QALY and $163 513/QALY, respectively, which were higher than our findings of $128 753/QALY and $19 562/QALY. This discrepancy is primarily attributable to the mean hospitalization costs used: our model used a more recent estimate of $20 566, compared with $11 487 used by Hutton et al,^59,60^ reflecting updated data adjusted for GA and payer type.^36,37^

While our analysis found MV to be more cost-effective than nirsevimab, an indirect comparison between the 2 studies by Hutton et al^59,60^ reported the opposite. Differences in drug prices, uptake assumptions, and efficacy estimates likely account for this divergence. In our analysis, drug costs were $272 for MV and $496 for nirsevimab, vs $295 and $445, respectively, in Hutton et al,^59,60^ as drug costs have changed.^42^ Higher uptake was assumed for nirsevimab—consistent with the United Kingdom–based model of Hodgson et al^62^—based on anticipated differences in delivery setting, whereas Hutton et al^59,60^ assumed equal uptake. Our estimate of MV hospitalization efficacy of 67.9% was derived through harmonized trial definitions, compared with 56.8% in Hutton et al.^59,60^ Like Hutton et al, who evaluated the cost-effectiveness of nirsevimab and MV separately by timing, we also found that administration earlier in the season was cost-effective.

We restricted our population to those eligible for MV, that is, mothers of infants born at a GA of 32 weeks or later during the RSV season, to allow us to compare MV and nirsevimab. Nirsevimab covers a broader population, including extremely preterm infants and those who are 1 to 8 months of age at the start of the RSV season.^48^ Hutton et al^59,60^ evaluated the use of nirsevimab for infants who are 1 to 8 months of age but did not further explore cost-effectiveness of nirsevimab in preterm infants (GA<32 weeks), a subgroup with markedly higher RSV-associated hospitalization costs—3 to 8 times greater than those in full-term infants—while Yu et al^61^ did. Yu et al^61^ showed that nirsevimab may be cost-effective at a threshold of $150 000/QALY compared with no intervention in premature infants, even at a price of $1962 per dose.

This study provides insight into the cost-effectiveness of MV and nirsevimab, offering useful data for clinical practice guidelines and public health policymakers. These agents are the first preventive options available for the 90% of the general infant population previously ineligible for palivizumab but remaining at risk for RSV. While both interventions reduce RSV-related morbidity, nirsevimab’s cost may limit its cost-effectiveness when compared with MV. These findings support incorporating MV into routine immunization schedules for infants born between October and January. At current pricing, nirsevimab could be reserved in situations where MV is not an option. However, cost-effectiveness depends on infection incidence, vaccine efficacy, and cost. Continuous surveillance with systems such as the New Vaccine Surveillance Network is critical to refine recommendations.

### Limitations

Our study has some limitations. Indirect benefits and adverse effects of immunization were not included. Indirect benefits such as herd immunity were not accounted for, as a disease transmission model was not used. However, the effects of these interventions are predominantly direct, as infants are not considered a significant epidemiological subgroup for RSV transmission.^20,29,30,31^ Correspondingly, Li et al^63^ found comparable outcomes for medically attended RSV cases and mortality when comparing static and dynamic models.^57^ However, family members may transmit RSV infections to infants, accounting for as many as 58% of cases in 1 study.^64^ If MV imparts sufficient immunity to mothers, this effect on transmission should be explored. However, the impact of these interventions on reducing transmission remains uncertain. We did not consider other indirect impacts, such as the need to increase bed capacity or to pay staff overtime, which may be important from a societal perspective. Adverse effects were excluded, as severe events were rare and unlikely to differ from placebo.^24,26^ Like all cost-effectiveness studies, our conclusions are shaped by the assumptions used in the base-case analysis, but the scenario analyses allow readers to explore other situations. A WTP threshold of $150 000/QALY was used, which is cited as a reasonable upper-bound value for US-based analyses.^47,55,56,65^ While a cost threshold of $150 000/evLYG was used, no validated standard exists for evLYG and remains a topic of future study. However, Campbell et al^47^ have stated that the same cost-effectiveness thresholds could be used for both QALYs and evLYG.^65,66^ A variety of WTP thresholds exists in the US, so readers may interpret the provided ICERs appropriate to their context.

## Conclusions

Our findings in this economic evaluation demonstrated that MV administration in the first 4 months of the RSV season and throughout the RSV season could be cost-effective. Although more clinically effective, nirsevimab was cost-effective compared with MV in only October and November. Intervention use may be optimized by using nirsevimab earlier in the season and MV later in the season. By providing monthly cost-effectiveness data, our analysis allows policymakers to evaluate different strategies for decreasing the RSV burden.

## Notes

Supplement 1. **eTable 1.** Impact Inventory **eTable 2.** Model Parameters Explained **eFigure 1.** Annual Distribution of RSV Hospitalizations by Month **eTable 3.** RSV Infection Incidence by Age **eTable 4.** Immunization Efficacy Days Post Dose **eTable 5.** Lifetime Utility Values **eTable 6.** Model Estimates of a QALY-Based Cost-Effectiveness Analysis Associated With Nirsevimab **eTable 7.** Model Estimates of an evLYG-Based Cost-Effectiveness Analysis Associated With Infant and Maternal Immunization **eTable 8.** Model Estimates of a QALY-Based Cost-Effectiveness Analysis Associated With Infant and Maternal Immunization Using Median Health Care Utilization Costs **eTable 9.** Model Estimates of a QALY-Based Cost-Effectiveness Analysis Associated With Infant and Maternal Immunization Using the Friction Cost Method **eTable 10.** Model Estimates of a QALY-Based Cost-Effectiveness Analysis From a Health Care Perspective **eFigure 2.** Maternal Vaccine vs No Intervention Tornado Diagrams for a QALY-Based Analysis From a Societal Perspective for October Through December **eFigure 3.** Maternal Vaccine vs No Intervention Tornado Diagrams for a QALY-Based Analysis From a Societal Perspective for January, February, and Multicohort **eFigure 4.** Nirsevimab vs Maternal Vaccine Tornado Diagrams for a QALY-Based Analysis From a Societal Perspective for October Through December **eFigure 5.** Nirsevimab vs Maternal Vaccine Tornado Diagrams for a QALY-Based Analysis From a Societal Perspective for January, February, and Multicohort **eMethods.** Calculating Immunization Efficacy
eReferences.

Supplement 2.
Data Sharing Statement
