# Supplementary material for: Optimizing Timing for Respiratory Syncytial Virus Prevention Interventions for Infants
Source: JAMA Netw Open. 2025 Jul 23;8(7):e2522779. doi: 10.1001/jamanetworkopen.2025.22779 (PMC12287852; doi:10.1001/jamanetworkopen.2025.22779)
Supplement: Supplement 1. — eTable 1. Impact Inventory eTable 2. Model Parameters Explained eFigure 1. Annual Distribution of RSV Hospitalizations by Month eTable 3. RSV Infection Incidence by Age eTable 4. Immunization Efficacy Days Post Dose eTable 5. Lifetime Utility Values eTable 6. Model Estimates of a QALY-Based Cost-Effectiveness Analysis Associated With Nirsevimab eTable 7. Model Estimates of an evLYG-Based Cost-Effectiveness Analysis Associated With Infant and Maternal Immunization eTable 8. Model Estimates of a QALY-Based Cost-Effectiveness Analysis Associated With Infant and Maternal Immunization Using Median Health Care Utilization Costs eTable 9. Model Estimates of a QALY-Based Cost-Effectiveness Analysis Associated With Infant and Maternal Immunization Using the Friction Cost Method eTable 10. Model Estimates of a QALY-Based Cost-Effectiveness Analysis From a Health Care Perspective eFigure 2. Maternal Vaccine vs No Intervention Tornado Diagrams for a QALY-Based Analysis From a Societal Perspective for October Through December eFigure 3. Maternal Vaccine vs No Intervention Tornado Diagrams for a QALY-Based Analysis From a Societal Perspective for January, February, and Multicohort eFigure 4. Nirsevimab vs Maternal Vaccine Tornado Diagrams for a QALY-Based Analysis From a Societal Perspective for October Through December eFigure 5. Nirsevimab vs Maternal Vaccine Tornado Diagrams for a QALY-Based Analysis From a Societal Perspective for January, February, and Multicohort eMethods. Calculating Immunization Efficacy eReferences. [file jamanetwopen-e2522779-s001.pdf]

Supplementary Online Content

Nguyen D, Lee H, Pavia AT, Nelson R, Samore M, Chaiyakunapruk N. Optimizing timing for respiratory syncytial virus prevention interventions for infants. *JAMA Netw Open.* 2025;8(7):e2522779. doi:10.1001/jamanetworkopen.2025.22779

eTable 1. Impact Inventory

eTable 2. Model Parameters Explained

eFigure 1. Annual Distribution of RSV Hospitalizations by Month

eTable 3. RSV Infection Incidence by Age

eTable 4. Immunization Efficacy Days Post Dose

eTable 5. Lifetime Utility Values

eTable 6. Model Estimates of a QALY-Based Cost-Effectiveness Analysis Associated With Nirsevimab

eTable 7. Model Estimates of an evLYG-Based Cost-Effectiveness Analysis Associated With Infant and Maternal Immunization

eTable 8. Model Estimates of a QALY-Based Cost-Effectiveness Analysis Associated With Infant and Maternal Immunization Using Median Health Care Utilization Costs

eTable 9. Model Estimates of a QALY-Based Cost-Effectiveness Analysis Associated With Infant and Maternal Immunization Using the Friction Cost Method

eTable 10. Model Estimates of a QALY-Based Cost-Effectiveness Analysis From a Health Care Perspective

eFigure 2. Maternal Vaccine vs No Intervention Tornado Diagrams for a QALY-Based Analysis From a Societal Perspective for October Through December

eFigure 3. Maternal Vaccine vs No Intervention Tornado Diagrams for a QALY-Based Analysis From a Societal Perspective for January, February, and Multicohort

eFigure 4. Nirsevimab vs Maternal Vaccine Tornado Diagrams for a QALY-Based Analysis From a Societal Perspective for October Through December

eFigure 5. Nirsevimab vs Maternal Vaccine Tornado Diagrams for a QALY-Based Analysis From a Societal Perspective for January, February, and Multicohort

eMethods. Calculating Immunization Efficacy

eReferences.

This supplementary material has been provided by the authors to give readers additional information about their work.

© 2025 Nguyen D et al. *JAMA Network Open*.

eTable 1. Impact Inventory

Sector	Type of Impact (list category within each sector with unit of measure if relevant)	Included in This Reference Case Analysis From…Perspective?		Notes on Sources of Evidence
		Health Care Sector	Societal	
**Formal Health Care Sector**				
Health	Health outcomes (effects)			
	Longevity effects	×	×	
	Health-related quality of life	×	×	
	Other health effects (e.g. adverse events and secondary transmissions of infections)			
	Medical costs			
	Paid for by third-party players	×	×	
	Paid for patients out-of-pocket			
	Future medical costs (payers and patients)			
	Future unrelated medical costs (payers and patients)			
**Informal Health Care Sector**				
Health	Patient-time costs			
	Unpaid caregiver-time costs		×	
	Transportation Costs	×	x	
**Informal Health Care Sector**				
Productivity	Labor market earnings lost		×	
	Cost of unpaid lost productivity due to illness		×	
	Cost of uncompensated household production		x	
Other (specify)				

N/A, not applicable

© 2025 Nguyen D et al. *JAMA Network Open*.

eTable 2. Model Parameters Explained

Parameters	Definitions	Additional Details
**General**		
Birth rate (per month)	Birth rate for full-term infants in the U.S.	A monthly birth rate 299,277 was estimated through published data from the National Vital Statistics System in 2023.[1] It was assumed that the birth rate remained constant from month to month. Early pre-term infants were excluded from this cohort.
Lifetime Expectancy (years)	Life expectancy for an individual in the U.S. birth	The life expectancy for the average individual in the United States estimated from the National Vital Statistics System in 2022 was used. [2]
RSV hospitalization incidence rate	Incidence of RSV hospitalization, which would include general and critical care admission	It was assumed that all hospitalizations are due to LRTIs. [3-5]Data from NVSN from 2016 to 2020 was used. NVSN collects data from 7 pediatric health systems located in Tennessee, New York, Ohio, Texas, Washington, Missouri, and Pennsylvania Incidence was age-adjusted and modeled seasonally and excluded early pre-term infants.
RSV outpatient infection incidence rate	Incidence of RSV outpatient-managed infections, which would include visiting outpatient care/emergency department but without hospitalization	Only LRTI- managed infections were included, as it was assumed that efficacy of these agents was limited to LRTI. This was calculated by assuming that 65% of all outpatient infections were due to LRTI.[3] Incidence was excluded early pre-term infants and was age-adjusted and modeled seasonally, with published data from the NVSN from 2005 to 2009. [3 6] The NVSN collects data from 7 pediatric health systems located in Tennessee, New York, Ohio, Texas, Washington, Missouri, and Pennsylvania.
**Clinical Parameters**		
Vaccination Uptake		
Maternal vaccine	Percentage of the population which would use maternal vaccine if universally offered.	Uptake was estimated from the national prenatal influenza vaccine uptake, which was taken from published data using the Prenatal Risk Assessment System (2012-2020)). [7] Prenatal influenza was used to estimate maternal vaccine uptake because the administration setting is similar (prenatal care).
Nirsevimab	Percentage of the population which would use nirsevimab if universally offered.	Uptake was estimated from birth-dose hepatitis B vaccine uptake, which was taken from published data using the National Immunization Survey (NIS) 2018-2019. [8] First-dose hepatitis B vaccine uptake was used to estimate nirsevimab uptake because the administration setting is similar (inpatient setting shortly after birth).

© 2025 Nguyen D et al. *JAMA Network Open*.

eTable 2. Model Parameters Explained

Immunization Hospitalization Efficacy		
Maternal vaccine	Vaccine efficacy against RSV-associated hospitalization for maternal vaccine	Hospitalization efficacy was used, but it was assumed that all hospitalizations were due to LRTIs. [9]
Nirsevimab	Vaccine efficacy against RSV-associated hospitalization for nirsevimab	Hospitalization efficacy for RSV-associated LRTI was used. [10]
Immunization Outpatient Efficacy		
Maternal vaccine	Vaccine efficacy against RSV-associated outpatient infection for maternal vaccine	It was assumed that maternal vaccine would only be effective against LRTI. Efficacy was calculated using clinical trial data for RSV-associated hospitalization and medically attended RSV-associated LRTI. [9] See supplemental methods for additional data.
Nirsevimab	Vaccine efficacy against RSV-associated outpatient infection for nirsevimab	It was assumed that nirsevimab would only be effective against LRTI. Efficacy was calculated using clinical trial data for RSV-associated hospitalized LRTI and medically attended RSV-associated LRTI. [10 11] See supplemental methods for additional data.
Duration of Illness (days)		
Hospitalization	The duration of hospitalization for RSV-associated illness, which would include general inpatient and outpatient	The average duration of hospitalization for full-term infants was used and estimated from Doucette et al. [12]
Post-Hospitalization	The duration post-hospitalization where the infant would still experience symptoms from illness	The average duration post-illness was estimated from Leidy et al. [13]
Outpatient infection	The duration of infection for which the infant would still experience symptoms	The average duration of symptoms was estimated from medical literature and previous cost-effectiveness analyses. [14-17]
Death		
Hospitalized RSV CFR	Death occurring during RSV due to hospitalization	The mortality rate during RSV-associated hospitalization was estimated from Doucette et al.[12] The mortality rate was weighted to account for high-risk and non-risk infants.[18]
Non-RSV Death Rate	Death occurring to non-RSV related causes	The non-RSV mortality rate infants was estimated using the National Vital Statistics system data from 2022. [19]

© 2025 Nguyen D et al. *JAMA Network Open*.

eTable 2. Model Parameters Explained

**Costs**		
Productivity Loss		
Caregiver salary (per work week)	Weekly salary for an average adult in the U.S.	The average salary for all occupations in the United States from the U.S. Bureau of Labor Statistics was used to calculate the average weekly salary. [20] A 40-hour work week was assumed, and the days of productivity loss was equivalent to the duration of infant illness.
Lifetime market productivity	Market productivity loss for an average individual in the U.S.	Market productivity loss was estimated using published data assessing [21]
Lifetime non-market productivity	Non-market productivity loss for an average individual in the U.S.	Non-market productivity consisted of other services providing societal value not captured in labor earnings: specifically, household, caring, and volunteer service. [21]
Medical Costs		
RSV hospitalization	Average cost of an RSV hospitalization in the U.S. for a full-term infant	The average cost of an RSV hospitalization in the U.S. for a full-term infant was estimated using published national cost data using Merative MarketScan databases.[22] The mean costs were used instead of the median, so that the costs for high-risk infants, i.e. those with chronic lung disease or chronic heart disease, were included.[23] The costs were weighted to according gestational age at birth and then calculated to reflect the U.S. payer mix. 15.5% of hospitalizations were assumed to occur in infants born at 32-36 wGa, and the remaining 75.5% were assumed to occur in full-term infants.[18] It assumed that 61% of hospitalizations were paid for by Medicaid and that 39% were paid for by commercial plans.[23]
Hospitalization transportation cost	Average cost of gas used to go the hospital in the U.S.	The average round-trip driving distance in the U.S. were 8.7 miles for hospital visits as reported by the Agency for Healthcare Research and Quality (AHRQ) Healthcare Cost and Utilization Project (HCUP).[24] The cost of gas was assumed to be $3.53 per gallon, and fuel efficiency was assumed to be 24.4 miles per gallon, according to the United States Department of Energy's 2024 estimates.[25 26]

© 2025 Nguyen D et al. *JAMA Network Open*.

eTable 2. Model Parameters Explained

RSV outpatient	Average cost of an RSV-associated outpatient infection in the U.S. for a full-term infant	The average cost of an RSV outpatient-managed infection in the U.S. for a full-term infant was estimated using published national cost data using Merative MarketScan databases.[22] The mean costs were used instead of the median, so that the costs for high-risk infants, i.e. those with chronic lung disease or chronic heart disease, were included.[23] The costs were weighted for according to gestational age at birth, site-of-care (emergency department or pediatric outpatient visit) and then calculated to reflect the U.S. payer mix. 5.8% of outpatient-managed infections were assumed to occur in infants born at 32-36 wGa, and the remaining 94.2% were assumed to occur in full-term infants.[6] 22.5% of outpatient-managed infections were assumed to be managed in the emergency department, and the remainder were assumed to be seen in the pediatric outpatient setting.[6] It assumed that 61% of infections were paid for by Medicaid and that 39% were paid for by commercial plans.[23]
Outpatient transportation cost	Average cost of gas used to go to the provider's office in the United States	The average round-trip driving distance in the U.S. was 10.04 miles for provider visits, as reported by data from the National Travel Surveys.[27] The cost of gas was assumed to be $3.53 per gallon, and fuel efficiency was assumed to be 24.4 miles per gallon, according to the United States Department of Energy's 2024 estimates.[25 26]
**Drug Costs**		
Maternal vaccine	Cost of maternal vaccine	Commercial and government vaccine prices were obtained from the CDC's Vaccine Price List, and a weighted average was then calculated based on the U.S. birth delivery payer mix.[28 29] It was assumed that 44.9% would be eligible for the government price and that 55.1% would receive the commercial price.
Nirsevimab	Cost of nirsevimab	Commercial and government vaccine prices were obtained from the CDC's Vaccine Price List, and a weighted average was then calculated based on the U.S. birth delivery payer mix.[28 29] It was assumed that 44.9% would be eligible for the government price and that 55.1% would receive the commercial price.
Administration cost	Cost for a provider to administer the immunization	The administration cost for injection was estimated from the Physician Services Fee schedule from 2024 [30].

© 2025 Nguyen D et al. *JAMA Network Open*.

eTable 2. Model Parameters Explained

**Utilities**		
RSV Hospitalization	Utility values during RSV hospitalization	Caregiver assessment of infant health and other modeled estimates of infant health during RSV infection was used. [13 15 31]
RSV Outpatient	Utility values during RSV-associated hospitalization	Caregiver assessment of infant health and other modeled estimates of infant health during RSV infection was used [13 15 31]
Lifetime	Lifetime utility values	For the QALY-based analysis, the utility values were estimated from Jiang et al, which gathered EQ-5D-5L data from the U.S. in a population-based survey. [32 33] For the evLYG-based analysis, the utility values were 0.851. [32 33]

CDC, Centers for Disease Control and Prevention; CFR, case fatality ratio; EQ-5D-5L, EuroQol 5 Dimensions 5 Level; evLYG, equal-value life years gained; LRTI, lower respiratory tract infection; QALY, quality-adjusted life year; RSV, respiratory syncytial virus; U.S., United States

© 2025 Nguyen D et al. *JAMA Network Open*.

eFigure 1. Annual Distribution of RSV Hospitalizations by Month

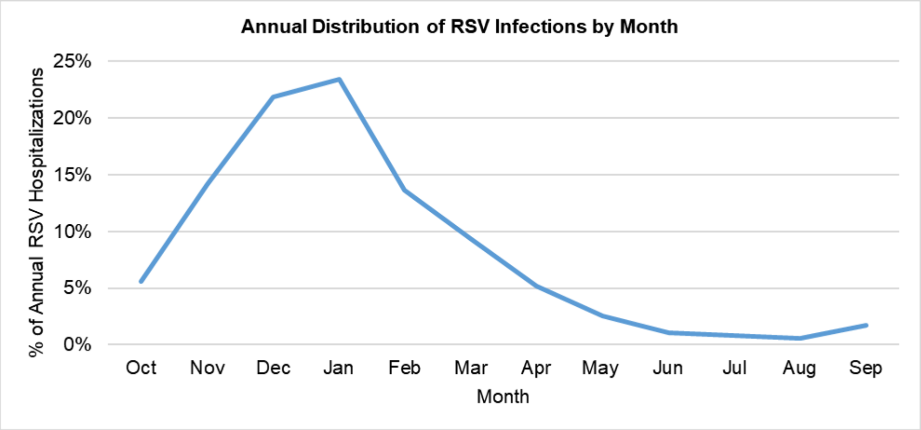

© 2025 Nguyen D et al. *JAMA Network Open*.

eTable 3. RSV Infection Incidence by Age

Month	Hospitalization Rate per 1000 Infants[34]<br>Mean (95% CI)	Outpatient Rate per 1000 Infants [6]<br>Mean (95% CI)
**0 months**	17.1 (14.8, 19.2)	64.4 (53.9, 74.8)
**1 month**	30.1 (27.5, 32.8)	154.9 (129.9, 179.7)
**2 months**	21.6 (19.6, 23.6)	188.3 (158.0, 218.7)
**3 months**	15.1 (13.5, 16.8)	207.5 (174.5, 240.6)
**4 months**	13.2 (11.6, 14.8)	234.0 (196.7, 271.4)
**5 months**	10.6 (9.2, 12.0)	221.4 (185.6, 257.3)
**6 months**	9.3 (7.7, 10.7)	105.2 (82.4, 114.1)
**7 months**	7.7 (6.2, 9.3)	91.0 (62.6, 86.7)
**8 months**	7.1 (5.9, 8.5)	74.5 (79.2, 109.9)
**9 months**	8.1 (6.6, 9.6)	94.5 (67.2, 93.1)
**10 months**	5.8 (4.6, 6.9)	80.0 (66.9, 93.0)
**11 months**	5.8 (4.6, 7.1)	88.9 (74.5, 103.4)

CI, confidence interval

© 2025 Nguyen D et al. *JAMA Network Open*.

eTable 4. Immunization Efficacy Days Post Dose

Days	Hospitalizations<br>Mean (95% CI)		Outpatient Infections[b]<br>Mean (95% CI)	
	**MV[9]**	**nirsevimab[10 11]**	**MV[9]**	**nirsevimab[10 11]**
30	0.679 (0.345, 0.843)	0.768 (0.494, 0.894)	0.482 (0.233, 0.650)	0.780 (0.569, 0.880)
60	0.679 (0.345, 0.843)	0.768 (0.494, 0.894)	0.482 (0.233, 0.650)	0.780 (0.569, 0.880)
90	0.679 (0.345, 0.843)	0.768 (0.494, 0.894)	0.482 (0.233, 0.650)	0.780 (0.569, 0.880)
120	0.315 (0, 0.707)	0.768 (0.494, 0.894)	0.482 (0.233, 0.650)	0.780 (0.569, 0.880)
150	0.315 (0, 0.707)	0.768 (0.494, 0.894)	0.482 (0.233, 0.650)	0.780 (0.569, 0.880)
180	0.315 (0, 0.707)	0.730 (0.469, 0.850)	0.482 (0.233, 0.650)	0.742 (0.567, 0.837)

CI = confidence interval; MV = maternal vaccine; RSV= respiratory syncytial virus
[a]The value for any hospitalization associated with RSV was used, not for lower-respiratory tract infection (LRTI) only.
[b]The value used was for non-severe medically-attended LRTI.
The lower bound was capped to 0

© 2025 Nguyen D et al. *JAMA Network Open*.

eTable 5. Lifetime Utility Values

Ages (years)	Utility	
	Mean	95% CI
**0 to 24**	0.919	(0.894, 0.943)
**25 to 34**	0.911	(0.897, 0.925)
**35 to 44**	0.841	(0.811, 0.872)
**45 to 54**	0.816	(0.782, 0.850)
**55 to 64**	0.815	(0.777, 0.853)
**65 to 74**	0.824	(0.786, 0.862)
**75+**	0.811	(0.767, 0.855)

CI, confidence interval

© 2025 Nguyen D et al. *JAMA Network Open*.

eTable 6. Model Estimates of a QALY-Based Cost-Effectiveness Analysis Associated With Nirsevimab

Analysis	Intervention	Total Costs, $	Total QALYs	Incremental Cost[a], $	Incremental QALYS[a]	ICER[a], Cost/QALY
**Healthcare Perspective**						
Oct	No Intervention	$122,679,162	146,929	Reference	Reference	Reference
	Nirsevimab	$171,347,427	147,185	$48,668,265	256	$190,236
Nov	No Intervention	$129,914,909	146,919	Reference	Reference	Reference
	Nirsevimab	$173,331,154	147,183	$43,416,245	264	$164,222
Dec	No Intervention	$114,711,944	146,980	Reference	Reference	Reference
	Nirsevimab	$166,706,909	147,209	$51,994,965	229	$226,961
Jan	No Intervention	$80,730,843	147,095	Reference	Reference	Reference
	Nirsevimab	$153,006,827	147,255	$72,275,984	160	$451,399
Feb	No Intervention	$49,089,466	147,199	Reference	Reference	Reference
	Nirsevimab	$140,583,358	147,296	$91,493,892	97	$945,295
Oct-Feb	No Intervention	$497,126,324	735,121	Reference	Reference	Reference
	Nirsevimab	$804,975,675	736,128	$307,849,351	1,006	$305,951
Societal Perspective						
Oct	No Intervention	$205,453,982	146,929	Reference	Reference	Reference
	Nirsevimab	$205,117,251	147,185	-$336,73	256	Dominant
Nov	No Intervention	$208,915,202	146,919	Reference	Reference	Reference
	Nirsevimab	$204,914,619	147,183	-$4,000,583	264	Dominant
Dec	No Intervention	$179,329,533	146,98	Reference	Reference	Reference
	Nirsevimab	$192,158,561	147,209	$12,829,028	229	$55,999
Jan	No Intervention	$124,456,363	147,095	Reference	Reference	Reference
	Nirsevimab	$170,027,957	147,255	$45,571,594	160	$284,617
Feb	No Intervention	$75,274,842	147,199	Reference	Reference	Reference
	Nirsevimab	$150,763,757	147,296	$75,488,915	97	$779,935
Oct-Feb	No Intervention	$793,429,922	735,121	Reference	Reference	Reference
	Nirsevimab	$922,982,14	736,128	$129,552,223	1,006	$128,753

ICER, incremental cost-effectiveness ratio; QALY, quality-adjusted life years

© 2025 Nguyen D et al. *JAMA Network Open*.

eTable 7. Model Estimates of an evLYG-Based Cost-Effectiveness Analysis Associated With Infant and Maternal Immunization

Analysis	Intervention	Total Costs, $	Total evLYG	Incremental Cost[a], $	Incremental evLYG[a]	ICER[a], Cost/evLYG
**Healthcare Perspective**						
Oct	No Intervention	$122,679,162	146,938	Reference	Reference	Reference
	Maternal vaccine	$139,387,889	147,070	$16,708,727	132	$126,240
	Nirsevimab	$171,347,427	147,188	$31,959,538	118	$270,571
Nov	No Intervention	$129,914,909	146,929	Reference	Reference	Reference
	Maternal vaccine	$140,605,776	147,073	$10,690,867	144	$74,164
	Nirsevimab	$173,331,154	147,187	$32,725,378	114	$286,762
Dec	No Intervention	$114,711,944	146,989	Reference	Reference	Reference
	Maternal vaccine	$129,175,278	147,117	$14,463,334	128	$113,007
	Nirsevimab	$166,706,909	147,213	$37,531,631	95	$393,087
Jan	No Intervention	$80,730,843	147,102	Reference	Reference	Reference
	Maternal vaccine	$106,805,364	147,192	$26,074,521	91	$287,215
	Nirsevimab	$153,006,827	147,258	$46,201,463	65	$707,730
Feb	No Intervention	$49,089,466	147,203	Reference	Reference	Reference
	Maternal vaccine	$86,645,441	147,258	$37,555,975	55	$677,057
	Nirsevimab	$140,583,358	147,297	$53,937,917	39	$1,388,471
Oct-Feb	No Intervention	$497,126,324	735,160	Reference	Reference	Reference
	Maternal vaccine	$602,619,748	735,711	$105,493,424	551	$191,546
	Nirsevimab	$804,975,675	736,143	$202,355,927	432	$468,583
**Societal Perspective**						
Oct	No Intervention	$205,453,982	146,938	Reference	Reference	Reference
	Maternal vaccine	$197,022,041	147,070	-$8,431,941	132	Dominant
	Nirsevimab	$205,117,251	147,188	$8,095,210	118	$68,534
Nov	No Intervention	$208,915,202	146,929	Reference	Reference	Reference
	Maternal vaccine	$194,596,896	147,073	-$14,318,306	144	Dominant
	Nirsevimab	$204,914,619	147,187	$10,317,723	114	$90,411
Dec	No Intervention	$179,329,533	146,989	Reference	Reference	Reference
	Maternal vaccine	$172,776,682	147,117	-$6,552,851	128	Dominant
	Nirsevimab	$192,158,561	147,213	$19,381,879	95	$202,996
Jan	No Intervention	$124,456,363	147,102	Reference	Reference	Reference
	Maternal vaccine	$136,032,856	147,192	$11,576,493	91	$127,517
	Nirsevimab	$170,027,957	147,258	$33,995,101	65	$520,749
Feb	No Intervention	$75,274,842	147,203	Reference	Reference	Reference
	Maternal vaccine	$104,054,075	147,258	$28,779,234	55	$518,831
	Nirsevimab	$150,763,757	147,297	$46,709,681	39	$1,202,402
Oct-Feb	No Intervention	$793,429,922	735,160	Reference	Reference	Reference
	Maternal vaccine	$804,482,550	735,711	$11,052,628	551	$20,068
	Nirsevimab	$922,982,145	736,143	$118,499,595	432	$274,402

[a]Maternal vaccine is being compared with no intervention; nirsevimab is being compared with maternal vaccine, the next best alternative.
evLYG, equal-value life years gained; ICER, incremental cost-effectiveness ratio; N/A, not applicable

© 2025 Nguyen D et al. *JAMA Network Open*.

eTable 8. Model Estimates of a QALY-Based Cost-Effectiveness Analysis Associated With Infant and Maternal Immunization Using Median Health Care Utilization Costs

Analysis	Intervention	Total Costs, $	Total QALY	Incremental Cost[a], $	Incremental QALY[a]	ICER[a], Cost/QALY
**Healthcare Perspective**						
Oct	No Intervention	$65,897,672	146,929	Reference	Reference	Reference
	Maternal vaccine	$100,869,113	147,064	$34,971,440	135	$258,421
	Nirsevimab	$148,248,222	147,185	$47,379,110	121	$393,176
Nov	No Intervention	$69,052,462	146,919	Reference	Reference	Reference
	Maternal vaccine	$101,138,023	147,067	$32,085,561	148	$217,041
	Nirsevimab	$149,010,053	147,183	$47,872,030	117	$410,763
Dec	No Intervention	$60,543,033	146,980	Reference	Reference	Reference
	Maternal vaccine	$94,772,862	147,111	$34,229,829	131	$260,353
	Nirsevimab	$145,309,791	147,209	$50,536,929	98	$517,702
Jan	No Intervention	$42,461,831	147,095	Reference	Reference	Reference
	Maternal vaccine	$82,817,975	147,188	$40,356,145	93	$432,382
	Nirsevimab	$138,017,176	147,255	$55,199,200	67	$826,573
Feb	No Intervention	$25,788,557	147,199	Reference	Reference	Reference
	Maternal vaccine	$72,142,519	147,256	$46,353,962	57	$812,613
	Nirsevimab	$131,474,061	147,296	$59,331,542	40	$1,492,781
Oct-Feb	No Intervention	$263,743,556	735,121	Reference	Reference	Reference
	Maternal vaccine	$451,740,493	735,686	$187,996,937	565	$332,731
	Nirsevimab	$712,059,303	736,128	$260,318,810	441	$590,035
**Societal Perspective**						
Oct	No Intervention	$148,672,493	146,929	Reference	Reference	Reference
	Maternal vaccine	$158,503,265	147,064	$9,830,772	135	$72,644
	Nirsevimab	$182,018,046	147,185	$23,514,782	121	$195,138
Nov	No Intervention	$148,052,755	146,919	Reference	Reference	Reference
	Maternal vaccine	$155,129,143	147,067	$7,076,387	148	$47,868
	Nirsevimab	$180,593,518	147,183	$25,464,375	117	$218,495
Dec	No Intervention	$125,160,622	146,980	Reference	Reference	Reference
	Maternal vaccine	$138,374,267	147,111	$13,213,645	131	$100,503
	Nirsevimab	$170,761,444	147,209	$32,387,177	98	$331,775
Jan	No Intervention	$86,187,350	147,095	Reference	Reference	Reference
	Maternal vaccine	$112,045,467	147,188	$25,858,117	93	$277,048
	Nirsevimab	$155,038,306	147,255	$42,992,838	67	$643,790
Feb	No Intervention	$51,973,933	147,199	Reference	Reference	Reference
	Maternal vaccine	$89,551,153	147,256	$37,577,220	57	$658,751
	Nirsevimab	$141,654,459	147,296	$52,103,306	40	$1,310,918
Oct-Feb	No Intervention	$560,047,153	735,121	Reference	Reference	Reference
	Maternal vaccine	$653,603,295	735,686	$93,556,142	565	$165,583
	Nirsevimab	$830,065,773	736,128	$176,462,478	441	$399,967

[a]Maternal vaccine is being compared with no intervention; nirsevimab is being compared with maternal vaccine, the next best alternative.
QALY, quality-adjusted life year; ICER, incremental cost-effectiveness ratio; N/A, not applicable

© 2025 Nguyen D et al. *JAMA Network Open*.

eTable 9. Model Estimates of a QALY-Based Cost-Effectiveness Analysis Associated With Infant and Maternal Immunization Using the Friction Cost Method

Analysis	Intervention	Total Costs, $	Total QALYs	Incremental Cost[a], $	Incremental QALYS[a]	ICER[a], Cost/QALY
**Societal Perspective**						
Oct	No Intervention	$191,505,104	146,929	Reference	Reference	Reference
	Maternal vaccine	$187,765,692	147,064	-$3,739,412	135	Dominant
	Nirsevimab	$199,627,907	147,185	$11,862,216	121	$98,439
Nov	No Intervention	$193,025,977	146,919	Reference	Reference	Reference
	Maternal vaccine	$184,521,676	147,067	-$8,504,301	148	Dominant
	Nirsevimab	$198,668,367	147,183	$14,146,691	117	$121,385
Dec	No Intervention	$164,712,113	146,980	Reference	Reference	Reference
	Maternal vaccine	$163,670,798	147,111	-$1,041,314	131	Dominant
	Nirsevimab	$186,431,217	147,209	$22,760,418	98	$233,158
Jan	No Intervention	$113,962,411	147,095	Reference	Reference	Reference
	Maternal vaccine	$129,568,299	147,188	$15,605,888	93	$167,204
	Nirsevimab	$165,932,394	147,255	$36,364,095	67	$544,529
Feb	No Intervention	$68,883,382	147,199	Reference	Reference	Reference
	Maternal vaccine	$100,148,568	147,256	$31,265,185	57	$548,098
	Nirsevimab	$148,277,896	147,296	$48,129,328	40	$1,210,933
Oct-Feb	No Intervention	$732,088,987	735,121	Reference	Reference	Reference
	Maternal vaccine	$765,675,032	735,686	$33,586,046	565	$59,443
	Nirsevimab	$898,937,780	736,128	$133,262,748	441	$302,052

[a]Maternal vaccine is being compared with no intervention; nirsevimab is being compared with maternal vaccine, the next best alternative.
ICER, incremental cost-effectiveness ratio; QALY, quality-adjusted life years

© 2025 Nguyen D et al. *JAMA Network Open*.

eTable 10. Model Estimates of a QALY-Based Cost-Effectiveness Analysis From a Health Care Perspective

Analysis	Intervention	Total Costs, $	Total QALYs	Incremental Cost[a], $	Incremental QALYS[a]	ICER[a], Cost/QALY
**Healthcare Perspective**						
Oct	No Intervention	$122,679,162	146,929	Reference	Reference	Reference
	Maternal vaccine	$139,387,889	147,064	$16,708,727	135	$123,469
	Nirsevimab	$171,347,427	147,185	$31,959,538	121	$265,216
Nov	No Intervention	$129,914,909	146,919	Reference	Reference	Reference
	Maternal vaccine	$140,605,776	147,067	$10,690,867	148	$72,318
	Nirsevimab	$173,331,154	147,183	$32,725,378	117	$280,798
Dec	No Intervention	$114,711,944	146,980	Reference	Reference	Reference
	Maternal vaccine	$129,175,278	147,111	$14,463,334	131	$110,008
	Nirsevimab	$166,706,909	147,209	$37,531,631	98	$384,475
Jan	No Intervention	$80,730,843	147,095	Reference	Reference	Reference
	Maternal vaccine	$106,805,364	147,188	$26,074,521	93	$279,366
	Nirsevimab	$153,006,827	147,255	$46,201,463	67	$691,838
Feb	No Intervention	$49,089,466	147,199	Reference	Reference	Reference
	Maternal vaccine	$86,645,441	147,256	$37,555,975	57	$658,379
	Nirsevimab	$140,583,358	147,296	$53,937,917	40	$1,357,077
Oct-Feb	No Intervention	$497,126,324	735,121	Reference	Reference	Reference
	Maternal vaccine	$602,619,748	735,686	$105,493,424	565	$186,710
	Nirsevimab	$804,975,675	736,128	$202,355,927	441	$458,657

[a]Maternal vaccine is being compared with no intervention; nirsevimab is being compared with maternal vaccine, the next best alternative.
ICER, incremental cost-effectiveness ratio; QALY, quality-adjusted life years

© 2025 Nguyen D et al. *JAMA Network Open*.

eFigure 2. Maternal Vaccine vs No Intervention Tornado Diagrams for a QALY-Based Analysis From a Societal Perspective for October Through December

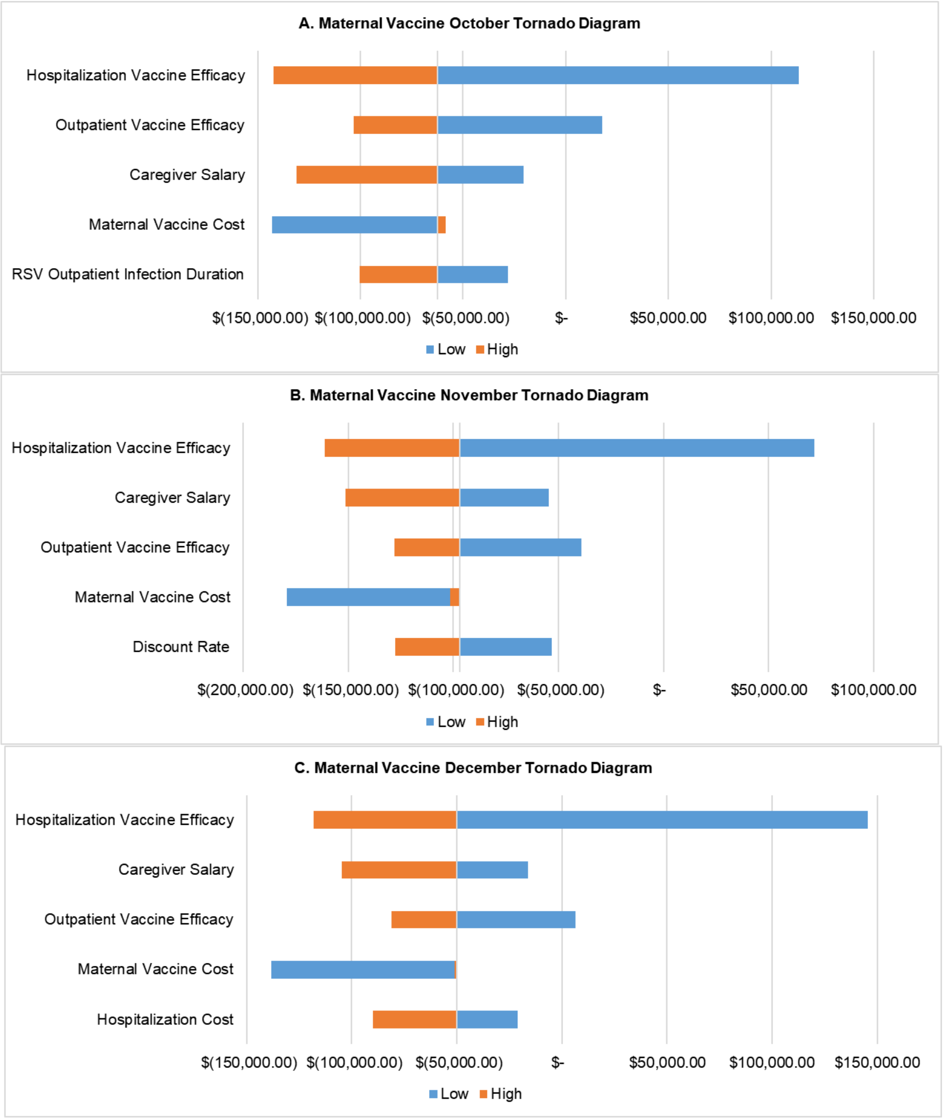

© 2025 Nguyen D et al. *JAMA Network Open*.

eFigure 3. Maternal Vaccine vs No Intervention Tornado Diagrams for a QALY-Based Analysis From a Societal Perspective for January, February, and Multicohort

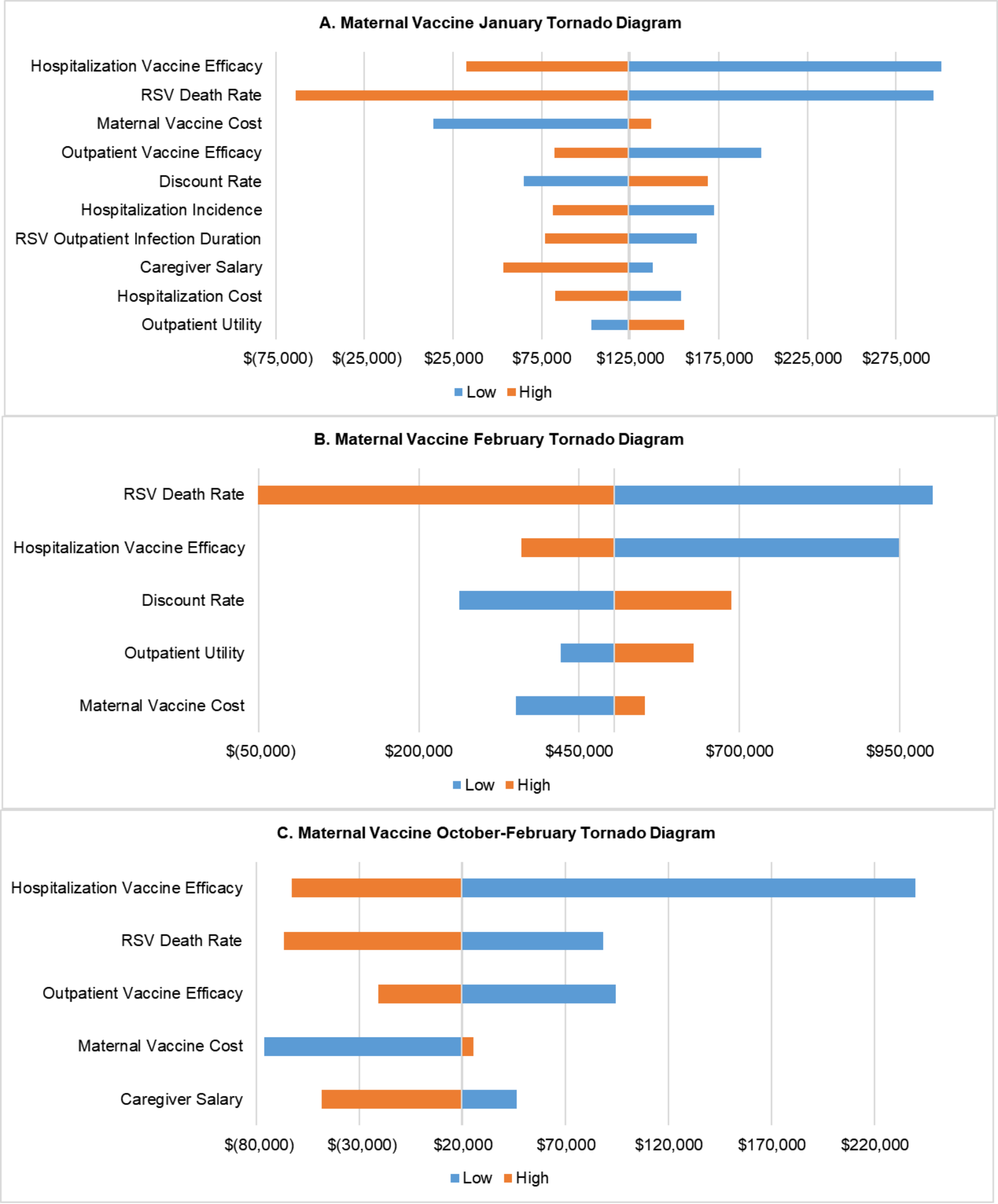

© 2025 Nguyen D et al. *JAMA Network Open*.

eFigure 4. Nirsevimab vs Maternal Vaccine Tornado Diagrams for a QALY-Based Analysis From a Societal Perspective for October Through December

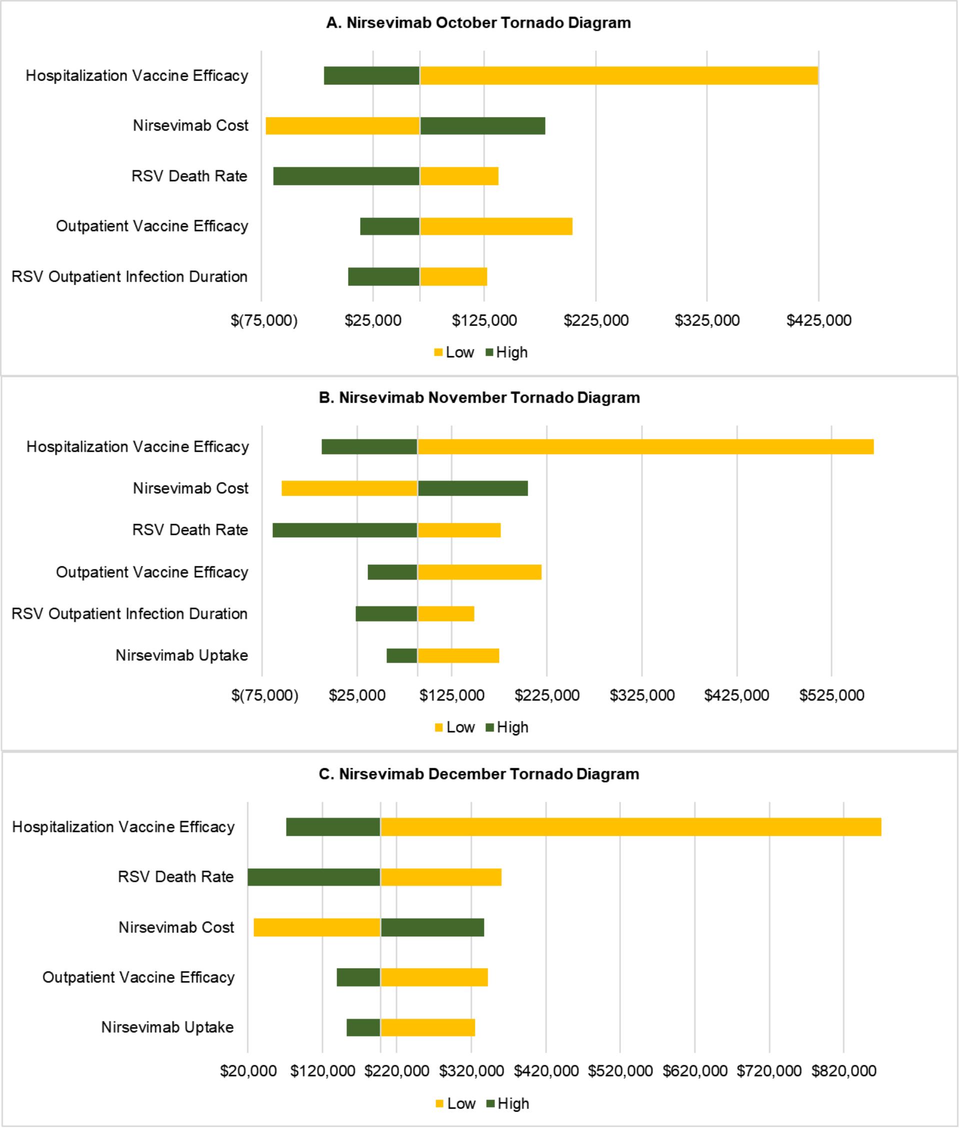

© 2025 Nguyen D et al. *JAMA Network Open*.

eFigure 5. Nirsevimab vs Maternal Vaccine Tornado Diagrams for a QALY-Based Analysis From a Societal Perspective for January, February, and Multicohort

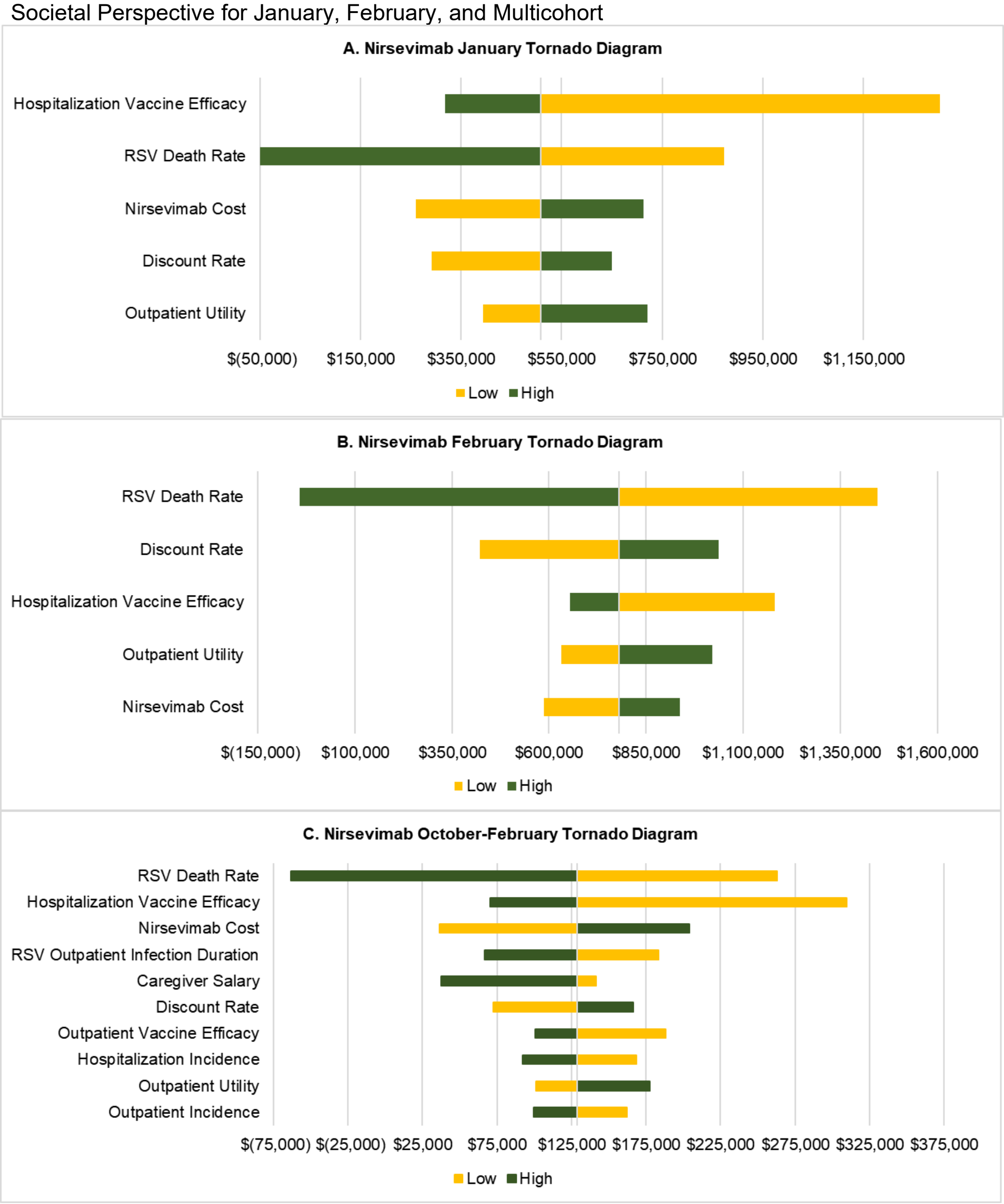

© 2025 Nguyen D et al. *JAMA Network Open*.

eMethods. Calculating Immunization Efficacy

1. **Estimating immunization efficacy**

Vaccine efficacy was calculated as:

$$VE = 1 - \frac{Incidence\ Rate\ for\ Immunization}{Incidence\ Rate\ for\ Placebo}$$

Immunization efficacy and its corresponding 95% confidence interval (CI) was calculated using a Poisson regression model. Vaccine efficacy was modeled using a monoexponential decay approach in R software, version 4.4.1 (R Foundation for Statistical Computing). Person-years were estimated using the time of follow-up. Aggregate count data from the MELODY and MATISSE studies were used as inputs. [9-11] It was assumed that RSV-associated hospitalizations were largely due to lower respiratory tract infections (LRTI). It was assumed that any "medically attended RSV-associated LRTI" infection that did not result in hospitalization would be managed in the outpatient setting. The number of "outpatient-managed RSV-associated LRTIs" was estimated by subtracting the "hospitalization for RSV-associated LRTI" events from the "medically attended RSV-associated LRTI" events.

2. **Extrapolating immunization efficacy beyond clinical trial data**

The time horizon utilized in this study was 180 days (6 months). While the MATISSE trial had immunization efficacy data for a year post-dose, the MELODY trial data were limited to the first 150 days post-dose, necessitating extrapolation for nirsevimab.[9-11] It was assumed that vaccine efficacy followed a mono-exponential decay function[35]:

$$VE_{T1} = VE_0 \times e^{-kt}$$

VE = vaccine efficacy
k = decay constant
t = days post-dose

Assuming that nirsevimab efficacy was 100% at time 0, the calculated k values for hospitalization and outpatient infections were estimated to be 0.0018 and 0.0017 respectively.

3. **Estimating the immunization efficacy for the 30-day cycle lengths**

The available immunization efficacies were for 0-90 days, 0-150 days, and 0-180 days, but as the cycle lengths in the model were for 0-30 days, 30-60 days, 60-90 days, 90-120 days, 120-150 days, and 150-180 days, the immunization efficacies for each of these cycles needed to be estimated.

By assuming that the infection incidence for placebo was constant, vaccine efficacy from time 1 (T1) to time 2 (T2) was approximated using the following equation:

$$VE_{0\text{-}T2} \approx VE_{T0\text{-}T1} \times \left(\frac{T1}{T2}\right) + VE_{T1\text{-}T2} \times \left(\frac{T2-T1}{T2}\right)$$

$$VE_{T1\text{-}T2} \approx \left[VE_{0\text{-}T2} - VE_{T0\text{-}T1} \times \left(\frac{T1}{T2}\right)\right] \times \left(\frac{T2}{T2-T1}\right)$$

VE = vaccine efficacy
k = decay constant
t = days post-dose
T1 = time 1
T2 = time 2

This led to the following results.

© 2025 Nguyen D et al. *JAMA Network Open*.

Immunization Efficacy by Cycle Length

Time Frame (Days)	Hospitalizations Mean (95% CI)		Outpatient Infections[b] Mean (95% CI)	
	**MV[9]**	**nirsevimab[10 11]**	**MV[9]**	**nirsevimab[10 11]**
0 to 30	0.679 (0.345, 0.843)	0.768 (0.494, 0.894)	0.482 (0.233, 0.650)	0.780 (0.596, 0.880)
30 to 60	0.679 (0.345, 0.843)	0.768 (0.494, 0.894)	0.482 (0.233, 0.650)	0.780 (0.596, 0.880)
60 to 90	0.679 (0.345, 0.843)	0.768 (0.494, 0.894)	0.482 (0.233, 0.650)	0.780 (0.596, 0.880)
90 to 120	0.315 (0, 0.707)	0.768 (0.494, 0.894)	0.482 (0.233, 0.650)	0.780 (0.596, 0.880)
120 to 150	0.315 (0, 0.707)	0.768 (0.494, 0.894)	0.482 (0.233, 0.650)	0.780 (0.596, 0.880)
150 to 180	0.315 (0, 0.707)	0.539 (0.347, 0.628)	0.482 (0.233, 0.650)	0.553 (0.422, 0.624)

CI = confidence interval; MV = maternal vaccine; RSV= respiratory syncytial virus
[a]The value for any hospitalization associated with RSV was used, not for lower-respiratory tract infection (LRTI) only.
[b]The value used was for non-severe medically-attended LRTI.

© 2025 Nguyen D et al. *JAMA Network Open*.

eReferences.

1. Martin JA, Hamilton BE, Osterman MJK. Births in the United States, 2023. NCHS Data Brief 2024(507) doi: 10.15620/cdc/158789.
2. Kochanek K. Mortality in the United States, 2022. Secondary Mortality in the United States, 2022 2024. https://www.cdc.gov/nchs/data/databriefs/db492.pdf.
3. Rainisch G, Adhikari B, Meltzer MI, Langley G. Estimating the impact of multiple immunization products on medically-attended respiratory syncytial virus (RSV) infections in infants. Vaccine 2020;**38**(2):251-57 doi: 10.1016/j.vaccine.2019.10.023 [published Online First: 20191116].
4. Curns AT, Rha B, Lively JY, et al. Respiratory Syncytial Virus-Associated Hospitalizations Among Children <5 Years Old: 2016 to 2020. Pediatrics 2024;**153**(3) doi: 10.1542/peds.2023-062574.
5. McMorrow ML, Moline HL, Toepfer AP, et al. Respiratory Syncytial Virus-Associated Hospitalizations in Children <5 Years: 2016-2022. Pediatrics 2024;**154**(1) doi: 10.1542/peds.2023-065623.
6. Lively JY, Curns AT, Weinberg GA, et al. Respiratory Syncytial Virus-Associated Outpatient Visits Among Children Younger Than 24 Months. J Pediatric Infect Dis Soc 2019;**8**(3):284-86 doi: 10.1093/jpids/piz011.
7. Jarshaw CL, Omoregie O, Peck JD, et al. Vaccination during pregnancy by race/ethnicity: a focus on American Indians/Alaska Natives. AJOG Glob Rep 2024;**4**(1):100318 doi: 10.1016/j.xagr.2024.100318 [published Online First: 20240209].
8. Hill HA, Chen M, Elam-Evans LD, Yankey D, Singleton JA. Vaccination Coverage by Age 24 Months Among Children Born During 2018-2019 - National Immunization Survey-Child, United States, 2019-2021. MMWR Morb Mortal Wkly Rep 2023;**72**(2):33-38 doi: 10.15585/mmwr.mm7202a3 [published Online First: 20230113].
9. Kampmann B, Madhi SA, Munjal I, et al. Bivalent Prefusion F Vaccine in Pregnancy to Prevent RSV Illness in Infants. N Engl J Med 2023;**388**(16):1451-64 doi: 10.1056/NEJMoa2216480 [published Online First: 20230405].
10. Muller WJ, Madhi SA, Seoane Nunez B, et al. Nirsevimab for Prevention of RSV in Term and Late-Preterm Infants. N Engl J Med 2023;**388**(16):1533-34 doi: 10.1056/NEJMc2214773 [published Online First: 20230405].
11. Hammitt LL, Dagan R, Yuan Y, et al. Nirsevimab for Prevention of RSV in Healthy Late-Preterm and Term Infants. N Engl J Med 2022;**386**(9):837-46 doi: 10.1056/NEJMoa2110275.
12. Doucette A, Jiang X, Fryzek J, Coalson J, McLaurin K, Ambrose CS. Trends in Respiratory Syncytial Virus and Bronchiolitis Hospitalization Rates in High-Risk Infants in a United States Nationally Representative Database, 1997-2012. PLoS One 2016;**11**(4):e0152208 doi: 10.1371/journal.pone.0152208 [published Online First: 20160406].
13. Leidy NK, Margolis MK, Marcin JP, et al. The impact of severe respiratory syncytial virus on the child, caregiver, and family during hospitalization and recovery. Pediatrics 2005;**115**(6):1536-46 doi: 10.1542/peds.2004-1149.
14. Eiland LS. Respiratory syncytial virus: diagnosis, treatment and prevention. J Pediatr Pharmacol Ther 2009;**14**(2):75-85 doi: 10.5863/1551-6776-14.2.75.
15. Regnier SA. Respiratory syncytial virus immunization program for the United States: impact of performance determinants of a theoretical vaccine. Vaccine 2013;**31**(40):4347-54 doi: 10.1016/j.vaccine.2013.07.024 [published Online First: 20130727].
16. Hodgson D, Atkins KE, Baguelin M, et al. Estimates for quality of life loss due to Respiratory Syncytial Virus. Influenza Other Respir Viruses 2020;**14**(1):19-27 doi: 10.1111/irv.12686 [published Online First: 20191018].
17. Hak SF, Venekamp RP, Wildenbeest JG, Bont LJ. Outpatient respiratory syncytial virus infections and novel preventive interventions. Curr Opin Pediatr 2024;**36**(2):171-81 doi: 10.1097/MOP.0000000000001323 [published Online First: 20231212].
18. Rha B, Curns AT, Lively JY, et al. Respiratory Syncytial Virus-Associated Hospitalizations Among Young Children: 2015-2016. Pediatrics 2020;**146**(1) doi: 10.1542/peds.2019-3611 [published Online First: 20200616].
19. Ely DM DAImitUS, 2022: Data from the period linked birth/infant death file. National Vital Statistics Reports; vol 73 no 5. Hyattsville, MD: National Center for Health Statistics. 2024. DOI: https://dx.doi.org/10.15620/cdc/157006.
20. May 2023 National Occupational Employment and Wage Estimates. Secondary May 2023 National Occupational Employment and Wage Estimates 2024. https://www.bls.gov/oes/2023/may/oes_nat.htm.
21. Grosse SD, Krueger KV, Pike J. Estimated annual and lifetime labor productivity in the United States, 2016: implications for economic evaluations. J Med Econ 2019;**22**(6):501-08 doi: 10.1080/13696998.2018.1542520 [published Online First: 20181115].

© 2025 Nguyen D et al. *JAMA Network Open*.

Formatted: Danish

22. Averin A, Law A, Shea K, Atwood M, Munjal I, Weycker D. Episodic Cost of Lower Respiratory Tract Illness due to Respiratory Syncytial Virus Among US Infants During the First Year of Life. J Infect Dis 2024;**230**(2):480-84 doi: 10.1093/infdis/jiad598.
23. Bowser DM, Rowlands KR, Hariharan D, et al. Cost of Respiratory Syncytial Virus Infections in US Infants: Systematic Literature Review and Analysis. J Infect Dis 2022;**226**(Suppl 2):S225-S35 doi: 10.1093/infdis/jiac172.
24. Weiss AJ PG, Roemer M. . Secondary 2021. https://hcup-us.ahrq.gov/reports/methods/MS2021-02-Distance-to-Hospital.jsp.
25. Average Fuel Economy by Major Vehicle Category. Secondary Average Fuel Economy by Major Vehicle Category. https://afdc.energy.gov/data/10310.
26. Average Annual Fuel Use by Vehicle Type Secondary Average Annual Fuel Use by Vehicle Type 2024.
27. Akinlotan M, Khodakarami N, Primm K, Bolin J, Ferdinand AO. Travel for medical or dental care by race/ethnicity and rurality in the U.S.: Findings from the 2001, 2009 and 2017 National Household Travel Surveys. Prev Med Rep 2023;**35**:102297 doi: 10.1016/j.pmedr.2023.102297 [published Online First: 20230623].
28. CDC. Current CDC Vaccine Price List. Secondary Current CDC Vaccine Price List 2024. https://www.cdc.gov/vaccines-for-children/php/awardees/current-cdc-vaccine-price-list.html.
29. Valenzuela CP, Osterman MJK. Characteristics of Mothers by Source of Payment for the Delivery: United States, 2021. NCHS Data Brief 2023(468):1-8.
30. Physician Fee Schedule. CMS.gov: Centers for Medicare & Medicaid Services.
31. Glaser EL, Hariharan D, Bowser DM, et al. Impact of Respiratory Syncytial Virus on Child, Caregiver, and Family Quality of Life in the United States: Systematic Literature Review and Analysis. J Infect Dis 2022;**226**(Suppl 2):S236-S45 doi: 10.1093/infdis/jiac183.
32. Jiang R, Janssen MFB, Pickard AS. US population norms for the EQ-5D-5L and comparison of norms from face-to-face and online samples. Qual Life Res 2021;**30**(3):803-16 doi: 10.1007/s11136-020-02650-y [published Online First: 20201006].
33. Campbell JD, Whittington MD, Pearson SD. An Alternative Measure of Health for Value Assessment: The Equal Value Life-Year. Pharmacoeconomics 2023;**41**(10):1175-82 doi: 10.1007/s40273-023-01302-6 [published Online First: 20230717].
34. Centers for Disease Control and Prevention (CDC). (2021). Respiratory Syncytial Virus (RSV) Surveillance. https://www.cdc.gov/surveillance/nrevss/rsv/index.html
35. Wilkins D, Yuan Y, Chang Y, et al. Durability of neutralizing RSV antibodies following nirsevimab administration and elicitation of the natural immune response to RSV infection in infants. Nat Med 2023;**29**(5):1172-79 doi: 10.1038/s41591-023-02316-5 [published Online First: 20230424].

Formatted: Danish

Formatted: Danish

© 2025 Nguyen D et al. *JAMA Network Open*.
