# Supplementary material for: Optimizing Timing for Respiratory Syncytial Virus Prevention Interventions for Infants
Source: JAMA Netw Open. 2025 Jul 23;8(7):e2522779. doi: 10.1001/jamanetworkopen.2025.22779 (PMC12287852; doi:10.1001/jamanetworkopen.2025.22779)
Supplement: Supplement 2. — Data Sharing Statement [file jamanetwopen-e2522779-s002.pdf]

Data Sharing Statement

Nguyen. Optimizing Timing for Respiratory Syncytial Virus Prevention Interventions for Infants. *JAMA Netw Open*. Published July 23, 2025. doi:10.1001/jamanetworkopen.2025.22779

Data
Data available: No

Additional Information
Explanation for why data not available: All data were collected from publicly available resources (i.e. publications or databases) and were cited.
